# Increase in Incidence Rates and Risk Factors for Multidrug Resistant Bacteria in Septic Children: A Nationwide Spanish Cohort Study (2013–2019)

**DOI:** 10.3390/antibiotics12111626

**Published:** 2023-11-14

**Authors:** María Slocker-Barrio, Jesús López-Herce-Cid, Amaya Bustinza-Arriortúa, Elena Fresán-Ruiz, Iolanda Jordán-García, Juan Carlos de Carlos-Vicente, Elvira Morteruel-Arizcuren, Patricia García-Soler, Montserrat Nieto-Moro, Cristina Schüffelmann, Sylvia Belda-Hofheinz, Laura Ximena Herrera-Castillo, Sonia María Uriona-Tuma, Laia Pinós-Tella, Yolanda Peña-López, on behalf of the Pediatric-ENVIN-HELICS Study Group

**Affiliations:** 1 Pediatric Intensive Care Department, Hospital General Universitario Gregorio Marañón, 28009 Madrid, Spain; pielvi@hotmail.com (J.L.-H.-C.); amayabustinza@outlook.es (A.B.-A.); laura.herreracastillo@gmail.com (L.X.H.-C.); 2 Primary Care Interventions to Prevent Maternal and Child Chronic Diseases of Perinatal and Developmental Origin Network (RICORS), RD21/0012/0011, Instituto de Salud Carlos III, 28029 Madrid, Spain; 3 Gregorio Marañón Biomedical Research Institute, 28009 Madrid, Spain; 4 Mother and Child and Public Health Department, School of Medicine, Universidad Complutense de Madrid, 28040 Madrid, Spain; 5 Pediatric Intensive Care Unit, Hospital Sant Joan de Déu, 08950 Barcelona, Spain; elena.fresan@sjd.es (E.F.-R.); yolanda.jordan@sjd.es (I.J.-G.); 6 Immunological and Respiratory Disorders in the Pediatric Critical Patient Research Group, Institut de Recerca Sant Joan de Déu, 08950 Barcelona, Spain; 7 Consortium of Biomedical Research Network for Epidemiology and Public Health (CIBERESP), 28029 Madrid, Spain; 8 Pediatric Intensive Care Unit, Hospital Son Espases, 07120 Palma de Mallorca, Spain; juanc.decarlos@ssib.es; 9 Pediatric Intensive Care Unit, Hospital de Cruces, 48903 Bilbao, Spain; elvira.morteruelarizcuren@osakidetza.es; 10 Pediatric Intensive Care Unit, Hospital Regional de Málaga, 29010 Málaga, Spain; pagarsol79@gmail.com; 11 Pediatric Intensive Care Unit, Hospital Niño Jesús, 28009 Madrid, Spain; montsen@hotmail.com; 12 Pediatric Intensive Care Unit, Hospital La Paz, 28046 Madrid, Spain; cschuffelmann@yahoo.es; 13 Pediatric Intensive Care Unit, Hospital 12 de Octubre, 28041 Madrid, Spain; sylviabeldahofheinz@gmail.com; 14 Preventive Medicine and Public Health, ENVIN-HELICS Registry Administration, Hospital Universitari Vall d’Hebron, 08035 Barcelona, Spain; smuriona@vhebron.net (S.M.U.-T.); lpinos@vhebron.net (L.P.-T.); 15 Pediatric Intensive Care Department, Hospital Universitari Vall d’Hebron, 08035 Barcelona, Spain; yolanhcp@gmail.com; 16 Vall d’Hebron Institute of Research, 08035 Barcelona, Spain; 17 University of Texas Southwestern Medical Center, Dallas, TX 75235, USA

**Keywords:** drug-resistant bacteria, extended-spectrum beta-lactamase (ESBL), sepsis, PICU, surveillance

## Abstract

The emergence of multidrug-resistant (MDR) bacteria in children is a growing concern, particularly among septic patients, given the need for first-right dosing. Our aim was to determine the incidence rates and factors associated with MDR-sepsis in the pediatric intensive care unit (PICU), using data from the Spanish ENVIN-HELICS PICU registry between 2013 and 2019. The rate of MDR bacteria among septic children ranged between 5.8 and 16.2% throughout this study period, with a significant increase since 2015 (*p* = 0.013). MDR-gram-negative bacteria (92%), particularly EBL-Enterobacterales (63.7%), were the most frequent causative microorganisms of MDR-sepsis. During this study period, sixteen MDR-sepsis (32.6%) corresponded to intrahospital infections, and 33 (67.4%) had community-onset sepsis, accounting for 10.5% of the overall community-onset sepsis. Independent risk factors associated with MDR-sepsis were antibiotics 48 h prior to PICU admission (OR 2.38) and PICU onset of sepsis (OR 2.58) in >1 year-old children, and previous malnourishment (OR 4.99) in <1 year-old children. Conclusions: There was an alarming increase in MDR among septic children in Spain, mainly by gram-negative (ESBL-Enterobacterales), mostly coming from the community setting. Malnourished infants and children on antibiotics 48 h prior to PICU are at increased risk and therefore require closer surveillance.

## 1. Introduction

Sepsis is one of the leading causes of death in critically ill children, accounting for remarkably high mortality in all age groups regardless of geographic location and socioeconomic status and mainly affecting neonates and young children under 5 years of age [1]. Therefore, sepsis is a major driver of broad-spectrum antibiotic use and contributes to the emerging global threat of antimicrobial resistance. In turn, antimicrobial resistance negatively affects individuals with sepsis and contributes to the progression of infection to sepsis and septic shock by decreasing the effectiveness of available antimicrobial therapy. Currently, antimicrobial resistance in Gram-negative bacteria is a growing threat in Europe and worldwide [2,3]. The ability of these bacteria to acquire and modify new genetic material that confers on them new resistance mechanisms is a cause of great concern, especially in children, because of the more limited availability of antimicrobials in pediatric patients [4].

The choice of the empirical antimicrobial regimen in children with sepsis admitted to the Pediatric Intensive Care Unit (PICU) is a complex decision since prescribing physicians must weigh the need for broad-spectrum antibiotics with the concern about antimicrobial resistance. Although inadequate initial treatment in patients with septic shock decreases survival, inappropriate and unnecessarily prolonged use of broad-spectrum antimicrobials can also negatively influence morbidity and mortality in patients with sepsis [5,6,7]. This can negatively affect the course of sepsis since it facilitates the selection of resistant strains, increases *C. difficile* infections, and increases the need for long-term invasive devices, which can increase infectious complications and other adverse effects [8,9]. An update on MDR-sepsis epidemiology and on its associated risk factors among septic children would be helpful for physicians at the bedside.

The aim of this study was to analyze the incidence rate of sepsis caused by multidrug-resistant (MDR) microorganisms and describe the factors associated with MDR bacteria among septic children admitted to the PICU in a Spanish cohort. Our secondary objective was to determine its influence on outcomes.

## 2. Results

### 2.1. Study Population

**Figure 1:**
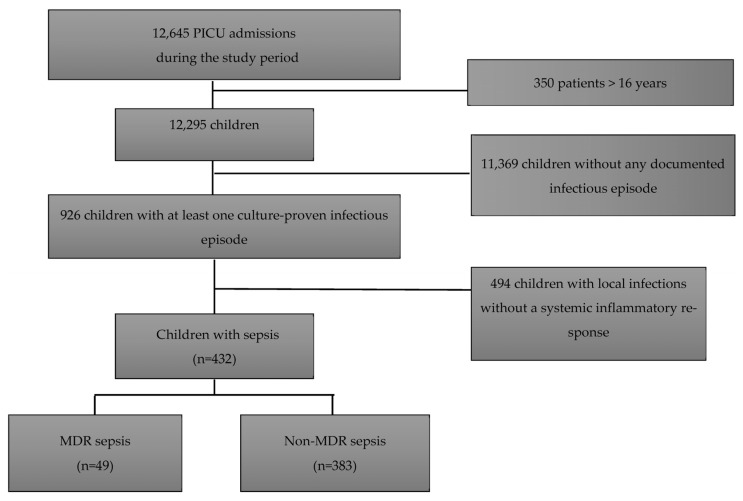
Study the flow chart.

During this study period, a total of 12,295 children were admitted to the PICU, of whom 926 had at least one culture-proven infectious episode (7.3%). The presence of an inflammatory systemic response following sepsis criteria was reported in 432 (47%) of them. Patients’ demographic characteristics are summarized in Table 1. Overall, patients with sepsis had a median age of 11 months [4–55 months] and a PRISM III score 24 h after admission of 7 [3,4,5,6,7,8,9,10,11,12]. One hundred and forty-one (32.6%) patients had received antibiotic treatment in the 48 h prior to PICU admission. Forty-nine culture-proven sepsis (11.3%) was caused by a MDR bacteria and 383 culture-proven sepsis (88.7%) by a non-MDR bacteria (Figure 1, Flow chart), with a larger proportion of Gram-negative bacilli (GNB) in the MDR group (*p* < 0.001). Among the 383 children with non-MDR sepsis, there were 111 (29%) patients with prior MDR infection/colonization.

### 2.2. Multidrug-Resistant Sepsis Rates, Overall Multidrug-Resistant Isolates, and Microorganism Profile over this Study Period

**Figure 2:**
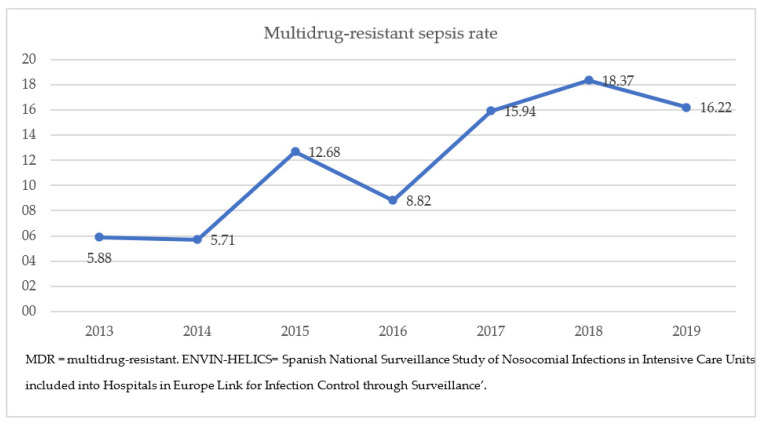
Evolution of the MDR sepsis rate in the pediatric ENVIN-HELICS registry, 2013–2019.

The rate of MDR bacteria among septic children ranged between 5.8 and 16.22% throughout this study period, with a significant increase since 2015 (*p* = 0.013) (Figure 2). ESBL-Enterobacterales were the most frequent causative microorganisms of MDR-sepsis (63.7% overall, *Klebsiella* spp. 36.7%), followed by *P. aeruginosa* spp. (16.3%), methicillin-resistant *S. aureus* (MRSA) (8%), *S. maltophilia* (6%), and other gram-negative bacteria (6%). Two *Klebsiella* spp. isolates produced metallobetalactamases, and one *P. aeruginosa* isolate was resistant to carbapenems.

**Figure 3:**
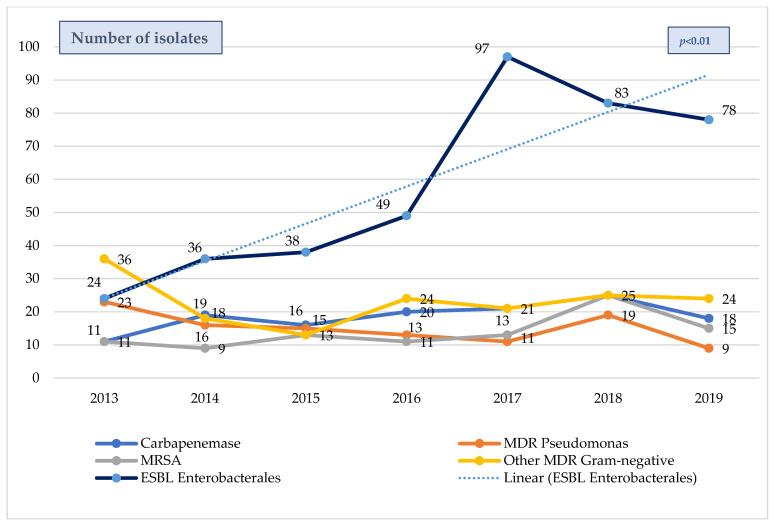
Evolution MDR bacteria isolates in septic patients from the pediatric ENVIN-HELICS registry 2013–2019. Includes colonization and infection. MDR = multi-drug-resistant. ESBL = extended spectrum β-lactamases. MRSA = methicillin-resistant *Staphylococcus aureus*. ENVIN-HELICS = Spanish National Surveillance Study of Nosocomial Infections in Intensive Care Units.

The evolution of the overall MDR bacteria isolates (colonization/infection) in PICU patients over the six-year pre-pandemic period (2013–2019) is shown in Figure 3. The most prevalent MDR microorganisms were Gram-negative, and the most frequent MDR species were also ESBL-Enterobacterales, which increased significantly throughout this study period (*p* < 0.01), while other species remained somewhat stable. The proportion of previously infected/colonized patients who developed sepsis due to a MDR bacteria was significantly higher than that of patients without previous colonization (30.6% versus 6.6%, *p* < 0.001), and the prevalence of MDR bacteria increased significantly with the severity of the inflammatory response (25.3% vs. 35.7% vs. 48.9%; non-inflammatory response, sepsis, and septic shock, respectively, *p* < 0.001).

### 2.3. Comparison between MDR and Non-MDR Septic Groups

Baseline overall and differential features between MDR and non-MDR sepsis patients are listed in Table 1. Compared to children with non-MDR sepsis, children with MDR sepsis were younger and had more frequent previous chronic diseases and malnutrition. These patients also received more frequent antibiotics 48 h prior to admission and total parenteral nutrition. There were no statistically significant differences in the proportion of community-onset/intrahospital sepsis, malignancy, immunosuppression, or previous hospital days pre-PICU between both groups. Table 2 shows the differences in the multivariate analysis of risk factors for mortality depending on the age group. Independent risk factors associated with MDR-sepsis were the use of antibiotics 48 h prior to PICU (OR 2.38 [1.06–5.36]; *p* = 0.037) and PICU sepsis debut (OR 2.58 [1.07–6.26]; *p* = 0.037) in >1 year-old children, and previous malnourishment (OR 4.99 [1.11–22.4]; *p* = 0.037) in <1 year-old children.

Baseline overall and differential risk factors for mortality between MDR and non-MDR sepsis patients are listed in Table S1 of the Supplementary Materials.

The median PICU length of stay (overall sepsis) was 20 days [11,12,13,14,15,16,17,18,19,20,21,22,23,24,25,26,27,28,29,30,31,32,33,34,35,36], and their overall PICU mortality rate was 12.7%. PICU stay was longer in children with MDR-sepsis compared to children with non-MDR sepsis (25.5 vs. 19 days; *p* = 0.006), but there were not statistically significant differences in PICU mortality (16.3% vs. 12.2%; *p* = 0.423) between both groups. Septic shock (OR 6.03 [2.75–13.2]; *p* <0.001), immunosuppression (OR 3.69 [1.73–2.79]; *p* = 0.001), and PICU sepsis (OR 2.95 [1.16–7.47]; *p* = 0.022) were independent factors associated with mortality in overall sepsis. In septic MDR-colonized/Infected children (n = 160), immunosuppression (OR 3.4 [1.2–10.1]) and the presence of septic shock (OR 4.3 [1.3–14]) were independent risk factors for mortality. In those with MDR-sepsis, only septic shock (OR 50.7 [1.4–8]).

## 3. Discussion

This is the first epidemiologic study to assess MDR rates and variables potentially associated with MDR-sepsis among critically ill children with sepsis and septic shock in a large Spanish cohort. Despite its low rate, MDR-sepsis had a significant impact on PICU stays, although it showed no impact on mortality. In this cohort, the most frequent MDR species were ESBL-Enterobacterales, and this fact may have influenced our results due to a more favorable resistance profile and susceptibility to antimicrobials. Noteworthy, we found no difference in the proportion of intrahospital/community-onset MDR-sepsis compared to non-MDR-sepsis. However, we observed that > 48 h of antibiotics prior to PICU admission, PICU sepsis in children > 1 year-old, and malnourishment in children <1 year-old were associated with a higher risk for MDR microorganisms in septic critically ill children. These findings may have important clinical implications.

In our study, the median age of sepsis patients was 11 months, and almost half of the episodes occurred in children under 1 year old, indicating that, as described in the literature, a significant proportion of sepsis affects younger children aged below two years old [1,10]. Moreover, the median age of patients with sepsis caused by MDR bacteria was lower than sepsis caused by drug-susceptible microorganisms (8 vs. 12 months), even though only 22% of children were <1 year-old in the MDR-group vs. 49.3% in the non-MDR group. This may be explained by the great proportion of children affected by chronic conditions in the MDR-sepsis group.

A remarkable finding was that the proportion of intrahospital sepsis was not significantly different between both groups, even with gram-negative rods accounting for 92% of MDR microorganisms. Thus, even taking into consideration the low statistical power due to the small sample, these close values deserve our attention. Classically, intrahospital and community infections have been known to have different causative agents, and antibiotic guidelines for empiric antibiotic therapy were therefore written accordingly [11]. In our cohort, a high proportion of children with MDR-sepsis (67.4%) came from the community, although MDR bacteria were responsible for only 10.4% of community-onset sepsis. In the era of the global dissemination of antimicrobial resistance, risk factors for the likelihood of drug-resistant infection should be cautiously evaluated, especially in septic shock [12,13]. In view of our results, other factors than the classical categorization between intrahospital/community onset, immunosuppression, or chronic conditions should be considered [14,15]. Analysis of our data in children > 1 year-old found that prior antibiotic prescription and PICU onset of sepsis were factors associated with higher risk for MDR-sepsis, as previously reported by other studies [14,16]. Noticeably, in our cohort, antibiotic prescription 48 h prior to admission increased by more than 200% the risk for MDR bacteria when assessing septic patients in the PICU, independent of previous hospitalization for more than 10 days and chronic conditions, which imply contact with the health care settings. So, while other factors are predictors for both MDR colonization and infection [17,18], among septic patients admitted to the PICU, prior >48 h antibiotic administration would be the only independent factor for considering a MDR bacteria etiology, based on our results.

Immunosuppression and malignancy have also been reported to be associated with a higher risk for MDR sepsis and sepsis mortality [18,19], along with other chronic comorbidities [1,10,16,20,21,22]. In our cohort, chronic conditions and malignancy seemed to be subrogates of worse outcomes, probably linked to a higher risk of intra-PICU sepsis and septic shock in that subgroup of patients. Despite the fact that MDR pathogens have been independently associated with an increased risk of death irrespective of age and the etiology of sepsis [2,13], no significant differences were observed in the mortality rate between patients with or without MDR bacterial sepsis in our cohort. An important reason for this is the small sample size of the MDR-sepsis group, which in turn might be due to the optional reporting of MDR cases. The worse outcomes in MDR-sepsis reported by other studies can be explained by greater virulence (i.e., MRSA) [14], but mainly by other factors involving the early and appropriate treatment of sepsis, such as the increased toxicity, inadequate dosage, the greater need for invasive or surgical procedures, and especially the delay in the start of the appropriate antibiotic therapy [23,24]. Accordingly, we found that immunosuppression was associated with higher mortality in overall and MDR-colonized/infected patients, but that the only independent and strongest factor associated with a higher risk of mortality among MDR septic critically ill children was the presence of septic shock (OR 50.7). These results are in line with Greenberg et al. [25], who found that the odds of death from sepsis in immunocompromised patients depended more on differences in care delivery than immunosuppressive medical conditions in a large multicenter study.

Another interesting result was that malnourishment was a strong risk factor for MDR-sepsis in infants, regardless of other known risk factors for sepsis such as total parenteral nutrition therapy or the presence of chronic disease. Classically, malnutrition has been reported as a risk factor for infections in low- and middle-income countries; however, it also affects children with comorbidities and increases healthcare exposure in high-income countries [2,26,27]. Apart from its known role on host defense and risk for infection [30], the pathophysiological mechanisms through which childhood malnutrition and intestinal microbiota impact MDR infections have recently been reviewed by Holowka et al. [29]. Curiously, the most prevalent microorganisms amongst most studies examining the burden of MDR infections/carriage in malnourished sub-Saharan Africans were ESBL-Enterobacterales [30,31,32].

Our results show an increasing rate of MDR bacterial sepsis from 2015 onwards, with the most frequent MDR species being ESBL-Enterobacterales in this cohort. These data are consistent with those published by other authors, in which the incidence of severe infections and sepsis caused by MDR bacteria has increased regardless of socioeconomic status, even tripling in the US [33], but increasing as well in Europe [34], Asia [35], and África [31]. This upward trend has also been observed in other neighboring countries, where the prevalence of ESBL-Enterobacterales in bloodstream isolates in 2022 from children from Portugal (26–41%), Italy (42–50%), and Greece (70%) has consistently increased over the last 10 years. These numbers are considerably higher than those reported in Spanish children (20–27%), according to the European Antimicrobial Resistance Surveillance Network (EARS-Net) [36] and then those reported by other Spanish studies (10.7% and 2.6% in healthy infants (8–16 months) and Spanish schoolchildren (3–13 years old), respectively) [37,38]. Despite the significant increase in infection/colonization by ESBL-Enterobacterales, the overall number of carbapenemase-producing Gram-negative bacteria in our Spanish cohort remained stable during this study period, as reported by EARS-Net in 2022 (0–8%), in contrast to Portugal (4.7–10%), Italy (3.6–18.7%), and Greece (>70%) [36]. These data correspond to global bloodstream infections, as there are no available data regarding PICU sepsis from these countries to compare with the Spanish cohort. However, our data confirm an alarming shift towards gram-negative predominance among MDR bacteria as well as its increase among septic children, raising concerns about a future increase in carbapenemase-producing Gram-negative bacteria following other countries’ patterns in the near future.

The ARPEC (Antibiotic Resistance and Prescribing in European Children) study, carried out in 17 centers in 12 European countries over 3 years, collected 1441 isolates of resistant germs in blood cultures, among which 38.8% of *K. pneumoniae*, 29% of *P. aeruginosa*, and 23.9% of *E. coli* strains came from PICU [39]. Moreover, the presence of MDR bacteria in the community setting has also been noticed, to the point of considering them ubiquitous not only in the health environment but also in the community [40]. Najem et al. reported a 4.31% prevalence of MDR carriage in 3964 children screened prospectively for MDR at admission to a pediatric hospital in Germany, mainly gram-negative bacteria (3.64%), maybe reflecting the increase in children with multiple comorbidities, chronic diseases, and permanent invasive devices, but also other risk factors at home, including adults in contact with healthcare settings [41]. The impact of the COVID pandemic on our MDR Spanish PICU rates must be determined in the next few years.

Thus, in view of the above, standardization of MDR screening protocols and surveillance policies in the PICU is essential. Understanding local epidemiology and antimicrobial resistance patterns is key to adjusting the antibiotic regimen for patients with sepsis. This also raises an opportunity for the development of multidisciplinary antimicrobial stewardship programs to help improve the quality of antibiotic prescriptions and reduce the inappropriate use of antimicrobials in the PICU.

## 4. Strengths and Limitations

The main strength of our study is that it includes the largest and most complete registry regarding infections—including sepsis and septic—from patients admitted to 29 Spanish PICUs distributed throughout the national territory during the first 5 years of the ENVIN-HELICS study in children. Another advantage is that it includes different patient profiles and complexity, which makes it a varied and representative sample of the critically ill children in our country. Finally, this registry was prospectively defined and implemented with a risk-based approach.

Our study also has some limitations. First, our cohort was limited by the MDR microorganism incidence and by the 3-month per year full-reporting period, including risk factors. Given the low incidence of some of the events and risk factors, the power to demonstrate statistical differences was limited. Second, there is not a universal protocol requiring screening for MDR at PICU admission, and surveillance policies are not homogeneous among PICUs. Even so, our data also showed a significant increase in EBL colonization/infection rates. Third, this study was conducted in Spain, and the results may not be applicable to other settings due to the large variation in epidemiology, sepsis prevalence, case mix, and resource availability. Fourth, the usefulness of mortality as an outcome is limited since the impact of sepsis on survivors, who may present high morbidity and significant long-term complications and sequelae, cannot be evaluated. Finally, analysis of the pandemic and immediate post-pandemic period was not performed due to the high variability in PICU practices during the pandemics, including critically ill adult care, which could not be properly defined in the registry and may bias the results and data interpretation.

## 5. Materials and Methods

### 5.1. Study Design and Study Patients

This is an observational cohort study using data from the Spanish National Surveillance Study of Nosocomial Infections in Intensive Care Units included in the European registry ‘Hospitals in Europe Link for Infection Control through Surveillance’ (ENVIN-HELICS): an observational, prospective, multicentre, and nationwide study conducted in 29 PICUs and 219 adult ICUs in Spain during a 3-month period every year (from 1 April to 30 June). This registry provides relevant information about global infection rates (both community and HAI) in the PICU, including causative microorganisms and their antimicrobial resistance profiles. Data on all consecutively admitted patients are entered in a collaborative web database (http://hws.vhebron.net/envin-helics, accessed on 1 January 2023). Eligible subjects for this study were all children (≥1 month and ≤18-year-old patients) requiring PICU admission during this study period (2013–2019).

### 5.2. Data Collection

Variables collected for this analysis included demographic characteristics, Paediatric Risk of Mortality (PRISM III) score, comorbidities, diagnosis on admission, patient origin (community, hospital, other healthcare facilities), microbiological data, antibiotic susceptibility profile, and presence of inflammatory response and septic shock.

### 5.3. Study Definitions and Outcomes

Sepsis and septic shock were defined according to the Third International Consensus Definitions for Sepsis and Septic Shock [42]. Pediatric sepsis was defined as a life-threatening organ dysfunction caused by a dysregulated host response to infection, and septic shock is a subset of sepsis patients with cardiovascular dysfunction that persists after adequate fluid resuscitation or needs vasoactive drugs. Patients were classified as having septic shock if vasopressors were initiated/increased within 24 h of the blood culture collection. Community-onset infections were those diagnosed prior to or during the first 48 h of hospital admission. Immunosuppression was defined by the presence of at least one of the following medical conditions: active hematologic malignancy, transplantation, immunosuppressive therapy, chemotherapy in the 30 days before PICU admission, chronic systemic steroid therapy, and autoimmune disease. Malignancy was defined as an active solid or hematologic cancer disease. Multidrug resistance (MDR) is defined in the registry according to the international expert proposal by Magiorakos et al. [43] as non-susceptibility to at least one agent in three or more antimicrobial categories (extended-spectrum penicillins, carbapenems, cephalosporins, aminoglycosides, and fluoroquinolones for Gram-negative bacteria; and ampicillin, vancomycin, fluoroquinolones, fosfomycin, and linezolid for Gram-positive bacteria). To assess the incidence rate of MDR among septic patients, we divided the number of patients with MDR-sepsis by the total number of septic patients. Colonized/infected children by MDR microorganisms were defined as patients with at least one MDR microorganism isolated in at least one clinical sample prior to or during PICU admission. The primary outcome was to study the incidence rate and factors associated with MDR-sepsis in the PICU. Secondary outcomes were PICU mortality and length of stay.

### 5.4. Statistical Analysis

The statistical analysis was performed using IBM SPSS 25.0 Statistics^®^, IBM Corp. Released 2017, IBM SPSS Statistics for Windows, Version 25.0. Armonk, NY, USA: IBM Corp. Categorical variables are shown as frequency (n) and percentage (%), whereas quantitative variables are summarized as median and interquartile range [IQR] because they are not normally distributed. The comparison of qualitative variables was performed using the χ^2^–test or Fisher’s exact test. Continuous variables were compared using the Mann–Whitney U-test and Kruskal–Wallis-test when not normally distributed. Univariate logistic regression analyses were performed for MDR-sepsis and mortality. All clinically relevant variables and variables with *p* < 0.2 from the univariate analysis were included in the multivariate analysis using the stepwise method. Results were presented as *p*-values and odds ratios/regression coefficients with a 95% confidence interval. Probability values (P) of less than 0.05 were considered statistically significant.

## 6. Conclusions

MDR bacteria increased significantly among septic children throughout this study period. ESBL-Enterobacterales were the most frequently isolated, with more than two-thirds of MDR-sepsis corresponding to children admitted from the community. In clinical practice, antibiotic use >48 h prior to PICU admission and malnourishment in infants should be considered when assessing the risk for MDR-sepsis, especially with septic shock, regardless of the location of the patient at the sepsis onset. As MDR bacteria are a global emerging threat, more multicenter international studies are needed to assess their impact on pediatric critically ill patients and implement global surveillance bundles.

## Figures and Tables

**Table 1:** Baseline characteristics of this study population.

	TOTAL Sepsis (*n* = 432)	MDR Sepsis (*n* = 49)	Non-MDR Sepsis (*n* = 383)	*p*-Value
Male sex, *n* (%)	218 (50.5)	22 (44.9)	196 (51.2)	0.408
Age (months) [median, IQR]	11 [4–55]	8 [4–27]	12 [4–62]	<0.001
Age < 1 year-old, *n* (%)	200 (46.3)	11 (22.4)	189 (49.3)	<0.001
PRISM III score [median, IQR]	7 [3–12]	7 [3–12]	7 [3–12]	0.948
Type of patient at PICU admission, *n* (%)				
Medical	244 (56.5)	32 (65.3)	212 (55.3)	0.186
Non-medical	188 (43.5)	17 (34.7)	171 (44.7)	
Comorbidities				
Malignancy	39 (9)	3 (6.1%]	36 [9.4%]	0.601
Immunosuppression *	102 [23.6%]	9 [18.4%]	83 [21.7%]	0.901
Other chronic diseases **	108 [25%]	15 [30.6%]	50 [13%]	0.001
Hospital days pre-PICU [median, IQR]	1 [0–22.5]	2 [0–39]	1 [0–12]	0.654
>7 Hospital-days pre-PICU	154 (35.6%)	19 [38.7%]	123 [32.1%]	0.350
Antibiotics prior to PICU admission (48 h)	141 [32.6%]	23 [46.9%]	118 [30.8%]	0.023
Previous malnutrition	127 [29.4%]	26 [53.1%]	101 [26.4%]	<0.001
Previous surgery	106 [40.3%%]	31 [63.3%]	156 [40.7%]	0.591
TPN therapy	157 [36.3%]	24 [49%]	133 (34.4)	0.038
Intrahospital sepsis, *n* (%)	117 (27%)	16 (32.6)	100 (26.1)	0.330
Intra UCIP sepsis, *n* (%)	44 (10.2%)	10 (20.4%)	34 (8.9%)	0.012
Septic shock, (%)	45 (10.4%)	9 (18.4%)	36 (9.4%)	0.053
GNB sepsis, *n* (%)	89 (20.6%)	45 (91.8%)	44 (11.5%)	<0.001

Data are presented as median (interquartile range), *n* (%). PRISM III score = Pediatric Risk Mortality score; IQR = Interquartile range; Hospital days pre-PICU = hospital length of stay prior to PICU admission; PICU = pediatric intensive care unit; TPN = total parenteral nutrition; * Immunosuppression includes immune deficiency, neutropenia, transplant, and other forms of immune suppression; ** Chronic disease includes renal, liver, and lung disease; GNB = Gram-negative Bacilli.

**Table 2:** Multivariate analysis of risk factors for sepsis caused by MDR bacteria classified by age group.

	Age < 1 Year Old (*n* = 200)				Age >1 Year Old (*n* = 232)			
	OR	CI 95%		*p*	R	CI 95%		*p*
Septic shock	1.48	0.32	6.94	0.617	2.31	0.69	7.72	0.174
ATB 48 h prior	3.69	0.57	23.4	0.171	2.38	1.06	5.36	0.037
>10 hospital days pre-PICU	0.19	0.02	1.79	0.148	1.55	0.71	3.34	0.266
Type of patient at PICU admission	0.48	0.10	2.23	0.354	0.82	0.36	1.86	0.641
PICU sepsis	0.44	0.09	2.05	0.298	2.58	1.07	6.26	0.037
TPN	1.12	0.26	4.73	0.880	0.74	0.32	1.71	0.479
Previous malnutrition	4.99	1.11	22.4	0.037	0.53	0.29	1.23	0.140
Chronic illness	4.26	0.63	28.7	0.136	1.33	0.51	3.49	0.554
Malignancy	0.56	0.07	4.29	0.580	0.81	0.06	10.2	0.868
Immunosuppression	1.37	0.26	7.27	0.707	0.63	0.22	1.78	0.385

ATB = antibiotics. PICU = Pediatric Intensive Care Unit. TPN = total parenteral nutrition.

## Data Availability

Data are contained within the article and Supplementary Materials.

## Supplementary Materials

The following supporting information can be downloaded at: https://www.mdpi.com/article/10.3390/antibiotics12111626/s1, Table S1: Multivariate analysis of risk factors for mortality (Total septic population, MDR colonized and infected patients, and MDR septic patients).
