# Supplementary material for: Increase in Incidence Rates and Risk Factors for Multidrug Resistant Bacteria in Septic Children: A Nationwide Spanish Cohort Study (2013–2019)
Source: Antibiotics (Basel). 2023 Nov 14;12(11):1626. doi: 10.3390/antibiotics12111626 (PMC10669462; doi:10.3390/antibiotics12111626)
Supplement: Supplementary file 1 [file antibiotics-12-01626-s001.zip › Table S1 Multivariate Analysis Risk Factors for Mortality overall vs MDR-sepsis.pdf]

Table S1. Multivariate analysis of risk factors for mortality (total septic population, MDR colonized and infected patients and MDR septic patients).

	Total (n=432)				MDR colonized/Infected patients (N=160)			MDR-sepsis (n=49)			
**VARIABLE**	**OR**	**CI 95%**		***p***	**OR**	**CI 95%**	***p***	**OR**	**CI 95%**		***p***
Septic shock	**6.03**	**2.75**	**13.2**	**<0.001**	**4.32**	**1.33-13.9**	0.15	**50.7**	**1.41**	**80.03**	**0.031**
Age < 1 year	1.20	0.60	2.39	0.605	2.70	0.98-7.45	0.054	12.7	0.21	774.1	0.224
ATB 48 hours prior	1.54	0.71	3.33	0.272	0.75	0.32-2.83	0.944	5.13	0.27	97.63	0.276
MDR sepsis	0.72	0.26	2.01	0.538	0.96	0.26-2.20	0.604	-	-	-	-
>10 hospital days pre-PICU	1.31	0.64	2.64	0.457	1.22	0.43-3.45	0.701	1.11	0.04	30.57	0.951
Type of patient at PICU admission	0.56	0.27	1.15	0.114	0.54	0.18-1.61	0.267	0.05	0.01	2.516	0.138
PICU sepsis	**2.95**	**1.16**	**7.47**	**0.022**	2.45	0.63-9.57	0.196	36.9	0.61	223.7	0.085
TPN	1.34	0.83	3.40	0.124	1.49	0.49-4.50	0.478	0.31	0.01	6.311	0.443
Immunosuppression	**3.69**	**1.73**	**7.88**	**0.001**	**3.43**	**1.16-10.1**	**0.026**	2.66	0.09	77.74	0.569
Previous malnutrition	1.68	1.34	2.79	0.508	1.17	0.37-3.66	0.789	6.45	0.14	303.8	0.343
Chronic illness	2.06	0.92	4.64	0.080	1.97	0.65-5.93	0.228	5.96	0.17	206.3	0.324

ATB= antibiotic, PICU= pediatric intensive care unit, TPN= total parenteral nutrition, MDR= multidrug resistant, OR= odds ratio, CI= confidence interval.
